# Visual Features: Featural Strength and Visual Strength Are Two Dissociable Dimensions

**DOI:** 10.1038/srep13769

**Published:** 2015-09-08

**Authors:** Liqiang Huang

**Affiliations:** 1 Department of Psychology, The Chinese University of Hong Kong, Hong Kong, China.

## Abstract

Visual features are often assumed to be the general building blocks for various visual tasks. However, it is well known that some stimulus categories (i.e., basic features) can be processed in parallel, but others (e.g., Ts in different orientations) need to be scanned serially, and this difference in *featural strength* seems to be on a fundamentally different dimension from differences in *visual strength* (e.g., reduction in contrast). This study compared two *high-level* tasks, namely tasks that require a lot of attentional operations (change detection and pattern comparison), with one *low-level* task, namely a task that requires few attentional operations (perceptual discrimination). The results confirmed that featural strength has substantial effects on high-level tasks but only a negligible effect on the low-level task. The results also revealed a complementary interaction: Visual strength has a substantial effect on the low-level task, but a negligible effect on high-level tasks. Overall, featural strength and visual strength are two dissociable dimensions in processing of visual features. The present results, along with other findings, challenge the generality of processing visual features.

A common, though often implicit, assumption about visual features is that they are the *general* building blocks for different tasks. In other words, a subtler difference, when compared to a larger difference, is believed to be more difficult to perceive, more difficult to find when presented among other items, and more difficult to remember after the stimuli disappear. For example, a previous study found that across a range of features, the visual working memory capacities and search slopes for these features are perfectly correlated^1^. This seems to imply that performance on these tasks, when measured in appropriate ways, is exactly determined by a single dimension of *general strength* of featural differences.

Here, the term “visual features” refers to both basic features (e.g., colors, shapes) and non-basic features (e.g., Ts in different orientations). Consistent with this use, this previous study^1^ included both basic features and non-basic features, and their results indicated that the relationship found between working memory capacities and search slopes applies to both.

Following a previous report^2^, in the present study I look for deviations from the generality of visual features. In other words, I look for cases in which the relative efficiency of different feature dimensions depends on the task: Feature A may be processed more efficiently than Feature B in Task X but less efficiently in Task Y. In the previous report^2^, I found that the relative performance between bar orientations and colors differ dramatically in change detection and visual tasks and that this difference can be attributed to a spatial structure analysis mechanism for bar orientations. In the present study, I address another type of deviation, namely, the dissociation between *featural strength* and *visual strength* of visual features.

## Featural Strength and Visual Strength. 

Visual processing consists of an early stage, in which basic pieces of information are extracted in parallel, and a late stage, in which attentional focus serially selects subsets of information and gives conscious access to the selected subset. Feature integration theory^3^, for example, proposed an parallel stage, in which a set of basic features (e.g., color, shape, size) are extracted in parallel, followed by a serial stage, in which attention allows the binding of these features so that they can be consciously perceived as a whole.

In support of this parallel/serial distinction, studies have found that, in visual search tasks, an item that differs from others on a basic feature (e.g., color or shape) can be efficiently found regardless of the number of items, whereas an item that differs from others only in terms of how elements are spatially arranged (e.g., Ts in different orientations) has to be found through laborious serial scanning^4^. The general explanation for this is that a basic feature can be found because some early visual modules allow the observer to determine the locations associated with this feature^4^,^5^. However, the spatial arrangements of features are not registered in any module, so they can only be handled by a serial scan of focal attention^4^,^6^. For example, for the early visual modules, ┬, ⊢, ⊥, and ⊢ are registered in basically the same way (e.g., vertical bar, horizontal bar, cross), and the differences between them (i.e., how the vertical bar and the horizontal bar are spatially arranged) can only be handled by a one-by-one scanning of individual items.

The present study uses the term *featural strength* to describe what is different between these two types of items. In other words, stimulus types that can be efficiently processed are strongly coded as basic features, so we say that they score high on the dimension of featural strength. On the other hand, stimulus types that require laborious scanning are not coded as basic features so we say that they score low on the dimension of featural strength.

The quality of sensory representation of a stimulus can be strong or degraded, and this is a dimension that is potentially dissociable from featural strength. For example, a stand-alone item can turn from clearly visible to hardly visible if we reduce its contrast against the background, or embed it into noise. In the present study, this dimension is termed *visual strength*.

Are featural strength and visual strength of visual features two dissociable dimensions? On the one hand, previous studies seem to hold that a difference in featural strength reflects a fundamental distinction between categories of items that is dissociable from the stimulus items’ visual strength^3^,^4^. On the other hand, if there is only a single dimension of “general strength” for processing of visual features, then we would expect featural strength and visual strength to boil down to a single dimension. This study seeks to distinguish between these two possibilities.

For the present purposes, we need to find (a) stimulus categories that allow us to manipulate featural strength and visual strength and (b) tasks that can potentially show the dissociable effects of featural strength and visual strength. These will be introduced one by one.

### The Choices of Stimuli

**Figure 1:**
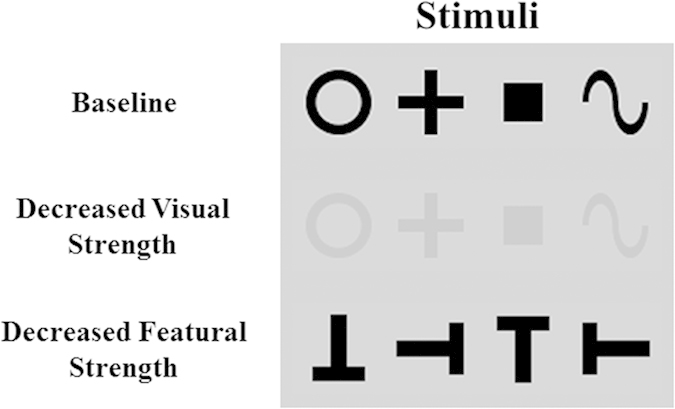
Examples of the stimuli used. The present experiments used three types of stimuli: (1) basic shapes serving as the “baseline” condition, (2) a low-contrast version of basic shapes for the “decreased visual strength” condition, and (3) Ts in different orientations for the “decreased featural strength” condition.

For manipulating the stimuli on the two dimensions of featural strength and visual strength, this study used three types of stimuli (Fig. 1): (1) basic shapes, (2) a low-contrast version of basic shapes, and (3) Ts in different orientations.

The stimulus type (1), as one of the most important basic features, is a classic example of parallel processing, whereas (3) is, to quote from a review paper summarizing decades of studies in visual attention^4^, “*a gold standard for ‘serial’ search.”* Therefore, a comparison between (1) and (3) provides a manipulation of featural strength. These specific stimulus types were chosen because they are widely used in the literature^4^,^6^.

Contrast is one of the most widely used manipulations in vision science to vary the visual strength of stimulus. Compared to the stimulus type (1), the contrasts of (2) have been reduced, but they are otherwise identical. Therefore, a comparison between (1) and (2) provides a manipulation of visual strength. Again, the manipulation of contrast is chosen because it is widely used in the literature^7^.

To reflect their roles in the present study, the three types of stimuli will be respectively termed the *baseline*, *decreased visual strength*, and *decreased featural strength* conditions.

### The Choices of Tasks

After determining the stimulus categories that allow us to manipulate featural strength and visual strength, we need to find tasks that can potentially show the dissociable effects of featural strength and visual strength. Specifically, we need a task that relies heavily on attentional operations and a task that does not. In this study, they will be termed a *high-level* task and a *low-level* task respectively. Since featural strength has a substantial impact on attentional processing, then we would naturally expect featural strength to have a substantial effect on high-level tasks, but a negligible effect on low-level tasks.

The low-level task chosen in this study was a perceptual discrimination task (i.e., to tell whether a target is the same as a cue). Perceptual discrimination is an unambiguous low-level task because only one item needs to be perceived, and therefore there is little need for attentional selections.

In this study, a high-level task is defined by laborious serial attentional operations, and the classic tasks requiring such attentional operations are inefficient visual search tasks (e.g., finding a T among other Ts in different orientations, as mentioned above^4^). However, the laborious nature of an inefficient visual search task is defined by the use of a specific set of stimuli rather than by the task itself. Thus, this task is *not* suitable for the present purposes. What is needed is a task in which even the basic features have to be scanned laboriously and serially. Two high-level tasks were chosen for this purpose: a pattern comparison task (i.e., to see whether two arrays of items are identical) and a change detection task. A pattern comparison of even basic features has been shown to be laborious^8^, and so this task was chosen for this study. A change detection task is also clearly a high-level task because, as a short-term memory test, attentional scanning is required to encode (or consolidate) the memory representations of each of the items^9^,^10^,^11^.

Analogously, the low-level and high-level tasks are similar to the “single item” and “many items” conditions respectively in a typical visual search experiment. The “single item” condition measures the situation when there is no need for serial attentional scanning, whereas the “many items” condition measures serial attentional scanning. A comparison between the two reveals the net effect of the attentional process.

In this study, the terms “high level” and “low level” are intended strictly in the way they are defined above—the presence/absence of laborious serial attentional operations. The use of these terms is, as far as I can tell, consistent with how they are commonly used in the area of visual attention. However, it should be mentioned that these umbrella terms have also been associated with many other concepts in the literature (e.g., automaticity), and I certainly do not intend to make generalized claims about them. I will revisit this issue in the general discussion.

## Effect of Visual Strength. 

As mentioned above, the tasks were chosen such that we expect featural strength to have a substantial effect on high-level tasks, but a negligible effect on low-level tasks. Then, what should we expect for visual strength? What seems certain is that the visual strength of the stimulus, which defines the quality of the “input data” that is used for the task, will have a substantial effect in a low-level task, which is a fairly direct processing of the input data.

**Figure 2:**
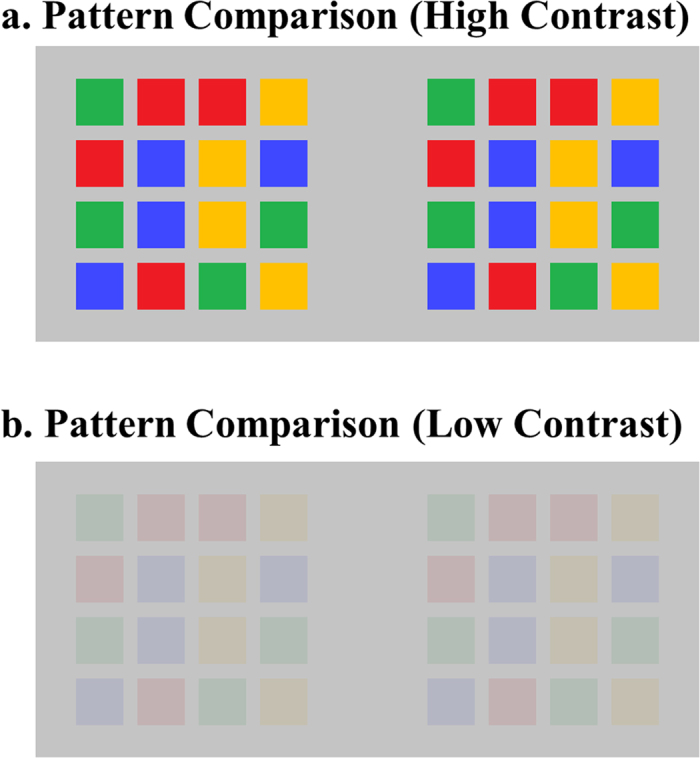
A research question. One question that was addressed in this study was whether *a high-level task is significantly affected by visual strength.* In the pattern comparison task, I ask whether this comparison is substantially more difficult in low-contrast displays (**panel b**) than in high-contrast displays (**panel a**).

However, to the best of my knowledge, no previous study has provided clear conclusions for the effect of visual strength in high-level tasks. The present study attempts to fill in this gap. To illustrate this question in a concrete task, consider the task of comparing two patterns of colors (Fig. 2), which is known to be a laborious task^8^: Here, I ask whether such a comparison will be substantially more difficult in low-contrast displays (Fig. 2b) than in high-contrast displays (Fig. 2a).

There seems to be no obvious answer to this question. On the one hand, it seems natural to predict that the performance of a high-level task would *hardly* be affected by visual strength because this task critically depends on high-level factors such as attentional operations. On the other hand, this pattern comparison task boils down to one-by-one comparisons of features, and each of these comparisons will become substantially more difficult when the stimulus strength is reduced. Therefore, it also seems natural to predict that the performance of this task would be *substantially* affected by visual strength. The present study attempts to distinguish between these two possible answers.

To sum up, there are two possibilities, neither of which seems implausible: (1) Visual strength has a substantial effect on a low-level task, but only a negligible effect on a high-level task; (2) Visual strength has substantial and comparable effects on both low-level and high-level tasks. What these two possibilities have in common is that the pattern of effects is different from that of featural strength. If there is only one single dimension of general strength, then any variation in the general strength of the stimuli should always have *proportional* effects on various tasks. Therefore, confirmation of *either* of the two possibilities will strongly suggest that the featural strength and visual strength are two dissociable dimensions. The single-dimension notion—namely that the featural strength and visual strength boil down to a single general strength—will only be supported if we find that visual strength has a substantial effect on a high-level task, but a negligible effect on a low-level task.

### The Choice of Measurements

In the present study, I compared the effect of featural strength and visual strength on the performance of three different tasks. One methodological challenge was that these differences may not be directly comparable: For example, is a difference of 10 ms in perceptual discrimination response time larger or smaller than a difference of 0.2 on estimated capacity (K) in change detection?

To solve this problem, in the perceptual discrimination and pattern comparison tasks, I measured exposure duration thresholds rather than response times because thresholds offer a consistent and comparable measure across different tasks. In addition, brief exposures exclude post-perceptual factors^12^,^13^ and generally give a purer and more direct measure of perceptual/attentional processing. Then the thresholds in these two tasks were compared along with the estimated capacity in change detection^14^,^15^, all on a logarithmic scale. Assuming the general applicability of Weber’s Law, it seems reasonable to consider 10% changes in threshold or capacity as equivalent (i.e., proportional) effects across these tasks.

## Experiment 1–3: Featural Strength and Visual Strength Are Two Dissociable Dimensions

### Method

#### Participants

University undergraduate students, all of whom had normal or corrected-to-normal vision, participated in this study’s experiments. Four participants (one in the perceptual discrimination task, one in the pattern comparison task, and two in the change detection task) were excluded because their performances were beyond predetermined criteria (average logarithmic threshold > Mean + 2 SD for pattern comparison and perceptual discrimination; average accuracy <0.55 for change detection). Other than these excluded participants, thirty-two participants took part in each of the tasks (i.e., perceptual discrimination, pattern comparison, change detection), making a total of 96 participants (64 women, mean age = 21 years).

All experiments were carried out in accordance with the approved guidelines. The consent form and experimental procedures received prior ethical approval from research ethics committee of the Chinese University of Hong Kong. Informed consent was obtained from each participant.

#### Apparatus

In all of the experiments, the stimuli were presented on a 1,024 × 768 pixel CRT monitor and the participants viewed the display from a distance of about 60 cm. The participants were asked to make responses by pressing one of two adjacent keys (“j” or “k”). They were asked to respond as accurately as possible but were under no time pressure (i.e., unspeeded responses).

#### Stimuli

Examples of the stimuli are shown in Fig. 1. I used three types of stimuli: (1) basic shapes (circles, squares, crosses, or squiggles) serving as the *baseline* condition, (2) low-contrast versions of basic shapes for the *decreased visual strength* condition, and (3) Ts in different orientations for the *decreased featural strength* condition. Each type of stimuli included four feature values.

**Figure 3:**
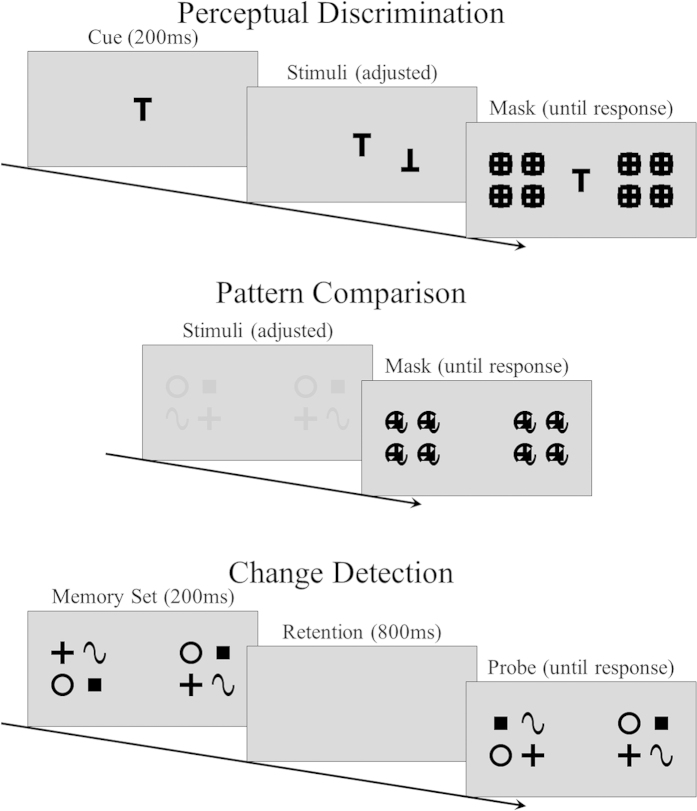
Sequence of presentations in the three tasks. In the perceptual discrimination task, the participants were asked to report whether a cue and a target were the same or not. In the pattern comparison task, the participants were asked to report whether the left and right sides of the stimuli display were the same or not. In the change detection task, the participants were asked to report whether the stimuli and the probe displays were the same or not.

As shown in Fig. 3, in the pattern comparison and change detection tasks, the stimulus items were presented in eight locations arranged as two 2 × 2 arrays on the left and right sides of the display and the centers of the arrays were 2.91 cm away from the center of the display. In each array, the distance between items, both horizontally and vertically, was 1.46 cm. In the perceptual discrimination task, the single item was randomly presented in one of the eight locations of the two arrays.

#### Procedure

The sequence of presentations is shown in Fig. 3. In all three tasks, a trial started with the presentation of a black fixation cross in the center of the display for 400 ms. This was followed by a gap of 400 ms, after which the stimuli display (or a cue) was presented (see below).

In the perceptual discrimination task (Experiment 1), a cue was presented and remained on the display. The stimulus display appeared 200 ms after the onset of the cue. Only one item (i.e., target) was presented in the stimuli display, and it was randomly placed on one of eight possible locations. The target was the same as the cue in 50% of the trials. The exposure duration of the stimuli display was adjusted for each individual participant to achieve a target accuracy of 75%. The stimuli display was followed by masks, which remained on the screen until a response was made. The participants were asked to report whether the cue and the target were the same or not.

In the pattern comparison task (Experiment 2), eight items were presented in the stimuli display, with each feature value used once on each side. The arrangement of the items was identical on the left and right sides in 50% of the trials; in the remaining 50%, two items on one side switched locations. The exposure duration of the stimuli display was adjusted for each individual participant to achieve a target accuracy of 75%. The stimuli display was followed by masks, which remained on the screen until a response was made. The participants were asked to report whether the left and right sides of the stimuli display were the same or not.

In the change detection task (Experiment 3), eight items were presented in the stimulus display, with each feature value presented once on each side and their arrangement randomized. The stimulus display (i.e., memory set) was presented for 200 ms, followed by a retention interval of 800 ms and then a probe display, which remained on the screen until a response was made. The probe display was identical to the stimulus display in 50% of the trials; in the remaining 50%, two items on the same side switched locations. The participants were asked to report whether the stimuli and the probe displays were the same or not.

In all three tasks, each participant completed seven blocks (96 trials per block); the first two blocks were treated as practice (and also as the “threshold adjustment” period) and excluded from the analysis. The three types of stimuli (baseline, decreased featural strength, decreased visual strength) were randomly intermixed within each block.

#### Results

**Figure 4:**
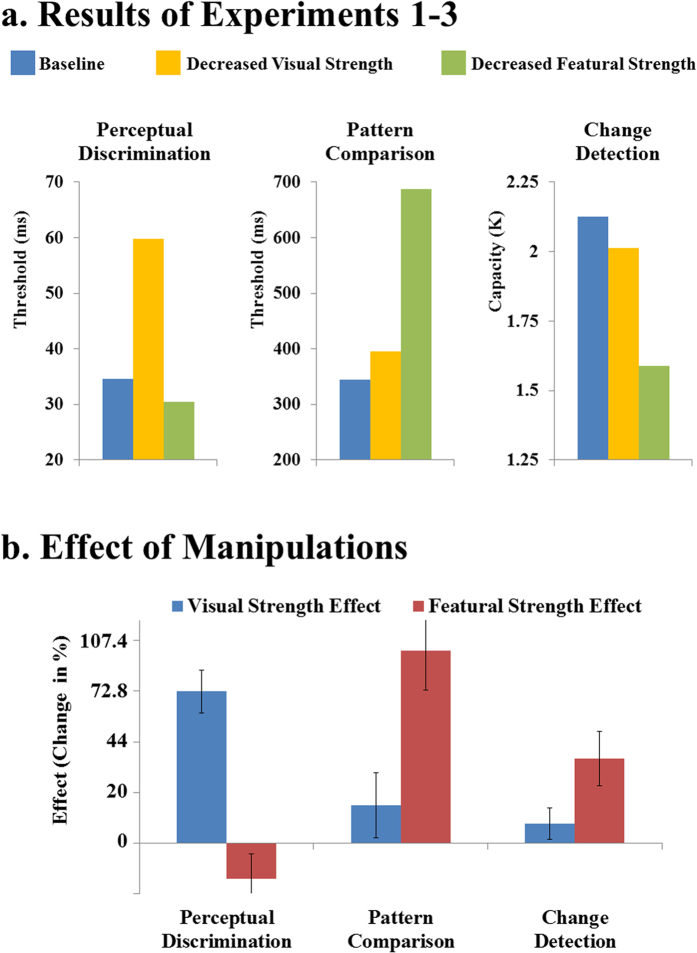
Results of Experiment 1–3. Panel (**a)** shows the results of Experiment 1–3. Panel (**b)** shows the effects of manipulations (i.e., visual strength effect vs. featural strength effect) and error bars show 95% confidence intervals. Visual strength had a substantial effect on the low-level task (perceptual discrimination) but only negligible effects on the high-level tasks (pattern comparison and change detection), whereas featural strength showed the opposite pattern: substantial effects on the high-level tasks, but only a negligible effect on the low-level task.

The results of Experiment 1–3 are plotted in Fig. 4a, and the effects of visual strength and featural strength (i.e., the differences between baseline and each of the other two conditions) are plotted in Fig. 4b. In the low-level task (perceptual discrimination), visual strength had a substantial effect, but featural strength had a slightly negative effect. The effects of featural and visual strengths were significantly different from each other (t (31) = 19.42; p < 0.0001; Cohen’s d = 3.43; BF_10 _= 6.89e + 15).

In the high-level tasks (pattern comparison and change detection), opposite patterns were found. Featural strength had substantial effects, but visual strength had very modest, and practically negligible, effects. The effects of featural and visual strengths were significantly different from each other (Pattern comparison: t (31) = 6.92; p < 0.0001; Cohen’s d = 1.22; BF_10 _= 160700; Change detection: t (31) = 4.59; p < 0.0001; Cohen’s d = 0.81; BF_10 _= 361). Across-group comparisons suggested that there were very large interactions between the effects (i.e., visual strength effect vs. featural strength effect) and the tasks (perceptual discrimination vs. pattern comparison: t (62) = 14.07; p < 0.0001; Cohen’s d = 3.52; BF_10 _= 4.58e + 17; perceptual discrimination vs. change detection: t (62) = 14.71; p < 0.0001; Cohen’s d = 3.68; BF_10 _= 3.64e + 18).

A potential criticism of the results of Experiment 1–3 is that the stimulus durations fell in very different ranges. Therefore, the different patterns of results may be caused by this difference on stimulus durations, rather than by the natures of tasks. Experiment 4–5 addressed this alternative account.

## Experiment 4–5: Ruling out the Effect of Stimulus Duration

To rule out stimulus duration as an alternative explanation for the results of Experiment 1–2, Experiment 4–5 repeated Experiment 1–2 but fixed the stimulus duration at constant levels and then used the accuracies of responses as the key measures for performances.

## Method. 

The methods of Experiment 4–5 were identical to that of Experiment 1–2 with the following exceptions. Eleven participants in the pattern comparison task were excluded because their performances were beyond a predetermined criterion (average accuracy < 0.55). Stimulus durations were fixed at 70 ms for perceptual discrimination and 100 ms for pattern comparison tasks. The duration for perceptual discrimination (Experiment 4, 70 ms) was determined by pilot testing with the goal of going up as much as possible but also staying far enough from the ceiling. The duration for pattern comparison (Experiment 5, 100 ms) was determined by pilot testing with the goal of going down as much as possible but also staying far enough from the floor.

## Results. 

**Figure 5:**
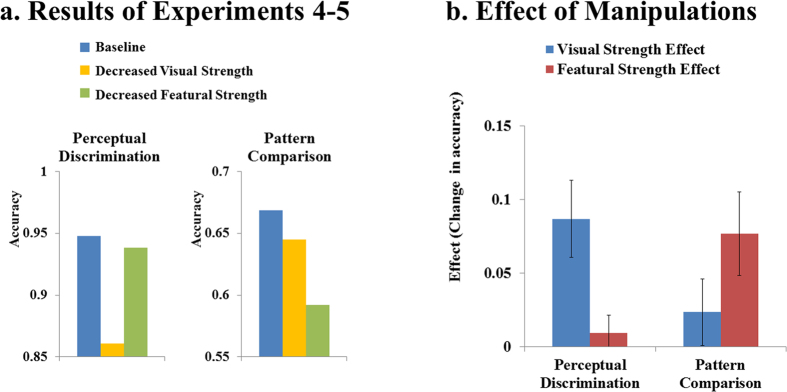
Results of Experiment 4–5. Panel (**a)** shows the results of Experiment 4–5. Panel (**b)** shows the effects of manipulations (i.e., visual strength effect vs. featural strength effect) and error bars show 95% confidence intervals. The pattern of results closely replicated that of Experiment 1–2. Visual strength had a substantial effect on the low-level task (perceptual discrimination) but only negligible effects on the high-level task (pattern comparison), whereas featural strength showed the opposite pattern: substantial effects on the high-level task, but only a negligible effect on the low-level task.

The results of Experiment 4–5 are plotted in Fig. 5a, and the effects of visual strength and featural strength are plotted in Fig. 5b. The pattern of results was clearly very consistent with those of Experiment 1–2. In the low-level task (perceptual discrimination), visual strength had a substantial effect, but featural strength had a negligible effect. The effects of featural and visual strengths were significantly different from each other (t (31) = 5.02; p < 0.0001; Cohen’s d = 0.89; BF_10 _= 1102). In the high-level tasks (pattern comparison), an opposite pattern was found. Featural strength had a substantial effect, but visual strength had a much smaller effect. The effects of featural and visual strengths were significantly different from each other (t (31) = 5.30; p < 0.0001; Cohen’s d = 0.94; BF_10 _= 2311). Across-group comparisons suggested that there was a very large interaction between the effects (i.e., visual strength effect vs. featural strength effect) and the tasks (perceptual discrimination vs. pattern comparison): t (62) = 7.10; p < 0.0001; Cohen’s d = 1.78; BF_10 _= 5694433.

Admittedly, Experiment 4–5 was imperfect in two ways. First, the duration of the pattern comparison task (100) was still longer than that of perceptual discrimination (70 ms). Second, the accuracies of perceptual discrimination and those of pattern comparison clearly were in different ranges. Unfortunately, these imperfections were difficult to avoid. A high-level task is, by definition, more complex and more difficult than a low-level task. Therefore, moving their stimulus durations to similar levels will unavoidably push the performance of the former toward a floor and that of the latter toward a ceiling.

While fully acknowledging these imperfections, Experiment 4–5 was unambiguous in showing the same stimulus-task interaction as in Experiment 1–2 when the durations of perceptual discrimination and pattern comparison were now made fairly similar to each other. Therefore, this stimulus-task interaction is unlikely to be due merely to a difference in durations.

## General Discussion

The results indicate that featural strength has substantial effects in high-level tasks, but not in low-level tasks, whereas the visual strength of features has substantial effects in low-level tasks, but not in high-level tasks. Overall, there is a clear double dissociation: Visual strength and featural strength are two dissociable factors that have substantial effects, respectively, on the processing of visual features in low-level and high-level tasks.

The results offered clear support for the idea that visual strength and featural strength are two dissociable dimensions. Certainly, this dissociation is very consistent with—or one might even say obvious from—what we have known about the distinction between basic features and other stimulus types that need to be found serially. The message of this study is to make an explicit and theoretically important point in showing this as a deviation from the generality of processing visual features.

Although the overall dissociation per se seems to be expected, the finding that visual strength has a substantial effect in a low-level task but not in high-level tasks, to the best of my knowledge, has addressed a novel empirical question, and the answer is, in my opinion, fairly surprising. To return to the concrete example in Fig. 2: Even though it seems intuitive that the comparison between color patterns should be substantially more difficult in Fig. 2b than in Fig. 2a, the results reveal only a relatively trivial difference.

### Featural Strength and Visual Strength

The difference between the featural strengths of different categories of stimuli has been a central trend in studies of visual attention. The original proposal was that there is a fundamental distinction between basic features and the binding of basic features^3^. However, subsequent studies have generally indicated that the binding of basic features can also be processed without focal attention^4^,^6^,^16^,^17^,^18^. On the other hand, the spatial arrangements of features (e.g., Ts in different orientations) still require focal attention^4^,^6^,^19^,^20^,^21^. The general explanation for this is that a basic feature can be found because some early visual modules allow the observer to determine the locations associated with this feature, whereas the bindings of basic features can be found by the joint use of multiple modules^4^,^5^. However, the spatial arrangements of features are not registered in any module and cannot be helped by combining multiple modules, so they can only be handled by a serial scan of focal attention^4^,^6^. The present study agrees with this consensus and takes the new perspective of considering this as a deviation from the generality of visual features in the hope of generating new insights, as will be discussed below.

To the best of my knowledge, no previous study has directly compared the role of visual strength in low-level and high-level tasks. In a broader context, the present results are clearly very relevant to the findings^22^,^23^,^24^, which suggest that the effect of visual salience is short lived. On a general level, the present study agrees with these previous studies on that, when stimuli are processed at a deeper level, bottom-up factors such as visual strength or visual salience no longer matter.

Why does visual strength matter only in low-level tasks and not in high-level tasks? As mentioned above, the general reason is probably that high-level tasks mainly depend on high-level factors such as attentional operations. Specifically, it seems plausible that visual strength determines the speed of feature extraction, but feature extraction is always a parallel process. Therefore, a reduction in visual strength leads to a relatively constant cost for performance, which does not increase proportionally with the length of the entire process. In the present study, this cost increased only moderately from 25 ms in perceptual discrimination to 51 ms in pattern comparison, whereas the threshold for baseline condition increased from 35 ms to 344 ms. Certainly, this relatively constant cost makes a substantial difference to perceptual discrimination tasks, in which the subsequent processing is easy, but makes (proportionally) little difference to high-level tasks in which the subsequent attentional operations are laborious. In a word, the present finding seems to suggest that a reduction in the visual strength of stimuli reduces the speed of feature extraction but does not weaken its parallel nature. This notion needs to be assessed further in future studies.

## High-Level and Low-Level Tasks. 

As stated above, in this study, the low-level/high-level distinction is only intended to describe the presence/absence of laborious attentional operations. However, in the literature, these umbrella terms have been associated with various other conceptual/empirical distinctions that do not always go along with each other^25^. For example, it is generally held that low-level processing is automatic and stimulus driven, whereas high-level processing is voluntary and goal driven^26^,^27^,^28^. Future studies will be needed to determine whether these other aspects (e.g., automaticity) interact with the effects of visual and featural strengths or not.

To avoid misinterpretation, it is also important to consider the “high-level” vs. “low-level” distinction in other research areas. For example, “high-level vision” sometimes refers to a highly specialized, algorithmically challenging visual function such as perceiving a face or a real-world scene. For the present purposes, these tasks would probably fall in the category of low-level tasks most of the time because these stimuli do not necessarily require more attention than simple artificial objects. On the other hand, some of the tasks that are considered low-level in other areas (e.g., the relatively simple games in problem solving) would probably fall in the category of high-level tasks for the present purposes because they require serial attentional operations.

It is also clear that, even only for how they are defined in this study, reality is not a dichotomy between purely high-level and purely low-level tasks but a continuum from tasks in which visual processing includes very little attentional operations to tasks in which visual processing depends more and more on attentional operations. Nevertheless, for the present purpose of showing the interaction between levels of processing and the different aspects of visual stimuli (i.e., visual strength vs. featural strength), it is sufficient to choose tasks that are at the two extremes of the continuum, like this study has done.

### Relation to another Dissociation

This study demonstrated the dissociation between visual strength, which is critical for low-level perceptual processing, and featural strength, which is critical for high-level attentional processing. In a broader context, this dissociation needs to be compared to the dissociation between central and input (visual) attention as revealed by previous studies^29^,^30^,^31^,^32^,^33^. These studies have shown that there is a “central” attentional limit that is required for processes such as making a decision or selecting a response and that this central attention is dissociated from visual attention.

Altogether, there seem to be three stages, the information of visual stimuli is first processed in a parallel perceptual stage, which is followed by serial visual attention, which selects a subset to reach conscious focus, which is then used in the central process for making a response. Although conceptually appealing, few previous studies have attempted to determine the relationships between all three stages in one single set of experiments. This needs to be explored in future studies.

### Challenge to the Generality of Visual Features

A common assumption about visual features is that these features are the general building blocks for various tasks. Consistent with this notion, a previous study^1^ found that, across a number of different features, the visual working memory capacities and search slopes for these features are perfectly correlated. The notion of the generality of visual features implies that the difference between features in various tasks can be attributed to a single dimension of *general strength*. One could say that, in that study^1^, visual search performance and the change detection performance are both merely manifestations of this general strength, and that is why they are perfectly correlated.

A natural prediction that follows from the notion of a single dimension of general strength is that any variation in the general strength of the stimuli should always have “proportional” effects on various tasks. This prediction is clearly undermined by the present finding that featural strength and visual strength have clearly disproportional effects on high-level and low-level tasks. Therefore, this study throws serious doubt on the generality of processing of visual features.

As mentioned above, the term “visual features” in this study refers to both basic features (e.g., shapes) and non-basic features (e.g., Ts in different orientations). One may potentially try to rescue the generality of visual features by limiting it to only basic features so that featural strength is no longer a “deviation.” But even for this more limited set, the disproportional effect of visual strength on a low-level and a high-level task is still a clear deviation from generality.

In a recent study, I reported another distinctive deviation from the generality of basic features^2^; this study was motivated by an attempt to analyze the spatial structure of features as characterized by the recent Boolean map notion that my collaborators and I proposed^8^,^9^,^34^. If a task requires the memory of a set of bars, then a Boolean map itself can be readily used to “hold” the overall structure and will be especially useful. If the task requires the visual search for a target, then the Boolean map, which contains only an overall structure, will hardly be useful. Therefore, bar orientations are processed more efficiently than colors in change detection tasks but less efficiently than colors in visual search tasks. This predicted interaction was confirmed: In comparison to the performance for colors, the performance for bar orientations were 40% better (on capacities) in a change detection task but 150% worse (on thresholds) in a visual search task.

To sum up, there are at least three separate dimensions that affect the processing of visual features: featural strength and visual strength as revealed in this study and the spatial structure analysis of features as revealed in a previous report^2^. A follow-up study has attempted to integrate them in one framework and to explore whether there are still other dimensions^35^. Complete knowledge of these deviations is important. On a theoretical level, it will reveal the fundamental relationships between various features and allow us to have a deeper understanding of the underlying mechanisms. Practically, it can help us to have better control over experiments. If one wants to control for feature differences in one study, one could arbitrarily use one particular task and jump to very different conclusions. A complete knowledge of these deviations would help avoid this problem.

## Additional Information

**How to cite this article**: Huang, L. Visual Features: Featural Strength and Visual Strength Are Two Dissociable Dimensions. *Sci. Rep.* **5**, 13769; doi: 10.1038/srep13769 (2015).
